# Identification of the *crp* gene in avian *Pasteurella multocida* and evaluation of the effects of *crp* deletion on its phenotype, virulence and immunogenicity

**DOI:** 10.1186/s12866-016-0739-y

**Published:** 2016-06-24

**Authors:** Xinxin Zhao, Qing Liu, Kangpeng Xiao, Yunlong Hu, Xueyan Liu, Yanyan Li, Qingke Kong

**Affiliations:** Institute of Preventive Veterinary Medicine, College of Veterinary Medicine, Sichuan Agricultural University, Chengdu, 611130 China; Avian Disease Research Center, College of Veterinary Medicine, Sichuan Agricultural University, Chengdu, Wenjiang, Sichuan 611130 China; Key Laboratory of Animal Disease and Human Health of Sichuan Province, Sichuan Agricultural University, Chengdu, Wenjiang, Sichuan 611130 China; Department of Bioengineering, College of Veterinary Medicine, Sichuan Agricultural University, Chengdu, Wenjiang 611130 China; State Key Laboratory of Food Science and Technology, Jiangnan University, Wuxi, 214122 China

**Keywords:** *Pasteurella multocida*, *crp*, Virulence, Regulated genes, Vaccine

## Abstract

**Background:**

*Pasteurella multocida* (*P. multocida*) is an important veterinary pathogen that can cause severe diseases in a wide range of mammals and birds. The global regulator *crp* gene has been found to regulate the virulence of some bacteria, and *crp* mutants have been demonstrated to be effective attenuated vaccines against *Salmonella enterica* and *Yersinia enterocolitica*. Here, we first characterized the *crp* gene in *P. multocida*, and we report the effects of a *crp* deletion.

**Results:**

The *P. multocida crp* mutant exhibited a similar lipopolysaccharide and outer membrane protein profile but displayed defective growth and serum complement resistance in vitro compared with the parent strain. Furthermore, *crp* deletion decreased virulence but did not result in full attenuation. The 50 % lethal dose (LD_50_) of the Δ*crp* mutant was 85-fold higher than that of the parent strain for intranasal infection. Transcriptome sequencing analysis showed that 92 genes were up-regulated and 94 genes were down-regulated in the absence of the *crp* gene. Finally, we found that intranasal immunization with the Δ*crp* mutant triggered both systematic and mucosal antibody responses and conferred 60 % protection against virulent *P. multocida* challenge in ducks.

**Conclusion:**

The deletion of the *crp* gene has an inhibitory effect on bacterial growth and bacterial resistance to serum complement in vitro. The *P. multocida crp* mutant was attenuated and conferred moderate protection in ducks. This work affords a platform for analyzing the function of *crp* and aiding the formulation of a novel vaccine against *P. multocida*.

**Electronic supplementary material:**

The online version of this article (doi:10.1186/s12866-016-0739-y) contains supplementary material, which is available to authorized users.

## Background

*Pasteurella multocida* (*P. multocida*) is a non-motile, capsulated, Gram-negative facultative anaerobic bacterium and is recognized as an important veterinary pathogen [1]. *P. multocida* is classified into five serogroups (A to F) based on its capsular antigens and into 16 serovars based on its somatic antigens [2]. Certain strains of *P. multocida* cause fowl cholera, a disease of poultry and wild birds resulting in high mortality rates with economic significance [3]. Some control is achieved with adjuvanted bacterins, which provide some degree of protective immunity and limit the incidence and severity of clinical disease, but this type of vaccine lacks the ability to induce long-term immunity and cross-protection against heterologous serotypes, resulting in immunized animals that continue to suffer disease outbreaks [4]. In an attempt to mimic natural infection and elicit long-term humoral and cellular immunity, empirically derived, live, avirulent strains have been developed. However, the basis for attenuation is not known, and reversion to virulence occurs [4]. Thus, new vaccines, particularly well-defined live vaccines, are required, and a significant amount of current research is directed toward achieving this goal.

The design of such vaccines is based on a wealth of new information on the pathogenesis of this bacterium. Global regulators play a vital role in the adaption of bacteria to the environmental changes that are encountered during infection, such as the PhoP/PhoQ regulators, which induce modifications of lipopolysaccharides (LPS) or outer membrane proteins (OMPs) to deal with environmental extremes and promote bacterial survival [5]. Many of these regulators are closely related to virulence [6–11] and are proven mutation targets for vaccine development [12, 13]. Crp (cAMP receptor protein) was the first prokaryotic transcription factor to be purified [14] and crystallized [15] from *Escherichia coli* (*E. coli*) and is the best characterized. Crp plays a vital role in the transcription of a series of genes for the utilization of carbon sources other than glucose [16]. This protein regulates the expression of numerous genes in response to variations in the intracellular concentration of cAMP [17], which is synthesized by membrane-bound adenylate cyclase. The *cya* gene, which encodes adenylate cyclase, is activated in the absence of glucose [18]. Upon binding to cAMP, the cAMP-Crp complex undergoes a conformational change that allows it to bind to promoters containing the consensus TGTGAN_6_TCACA sequence [19]. After binding to promoter DNA, Crp recruits RNA polymerase, resulting in the formation of specific protein-protein interactions that lead to the transcription of target genes. At some promoters, Crp also inhibits transcription via several mechanisms, such as promoter occlusion [20, 21]. A total of 254 target promoters have been identified in *E. coli* [22]. Crp has been shown to control the expression of essential virulence factors, and *crp* mutants attenuate the virulence of many Gram-negative bacteria, including *Salmonella enterica* [23], *Mycobacterium tuberculosis* [24], *Vibrio cholerae* [25] and pathogenic *Yersinia* species [26, 27]. Furthermore, *Salmonella enterica* strains with mutations in *crp* either alone or in combination with other genes have served as effective vaccine candidates against salmonellosis [28, 29].

The sequence and functions of the *crp* gene in *P. multocida* have not yet been identified. In this study, *P. multocida* 0818 was selected to investigate the putative *crp* gene. The bacterium was isolated from livers of ducks with a typical clinical representation of pasteurellosis from a duck farm suffering a pasteurellosis outbreak in southwest China. *P. multocida* 0818 was typed as capsular serotype A:1, nontoxinogenic, and was highly virulent, with a 50 % muscular lethal dose of <100 CFU being observed for 3-day-old ducklings (unpublished data). Here, the *crp* gene was first characterized from the virulent *P. multocida* 0818 strain. Then, the non-polar Δ*crp* mutant of *P. multocida* 0818 was constructed, and its phenotype, including its virulence, resistance to serum complement and bacterial growth, lipopolysaccharide (LPS) and outer membrane protein (OMP) profiles, were systematically investigated. *crp*-regulated genes were also identified through transcriptome sequencing. Moreover, the immunogenicity and protective efficacy of the Δ*crp* mutant were determined.

## Methods

### Bacterial strains, plasmids and growth conditions

The bacterial strains and plasmids used in this study are described in Table 1. *P. multocida* was grown at 37 °C in brain heart infusion (BHI) broth or on BHI agar (BD Bioscience, USA), and *Salmonella enterica* serovar Typhimurium (*S.* Typhimurium) and *E. coli* were grown in Luria-Bertani (LB) broth or on LB agar. When required, antibiotics and diaminopimelic acid (DAP) were added to the medium at the following concentrations: kanamycin, 50 μg/ml; ampicillin, 100 μg/ml; chloramphenicol, 25 μg/ml for *S.* Typhimurium and *E. coli* or 2.5 μg/ml for *P. multocida*; and DAP, 50 μg/ml [30]. The transformation of *S*. Typhimurium and *P. multocida* was performed via electroporation. Transformants were selected on LB or BHI agar plates containing appropriate antibiotics, and Asd^+^ plasmids were selected on LB agar plates.

**Table 1:** Bacterial strains and plasmids used in this study

Strains or plasmids	Description	Source
Plasmids		
pQK663	Asd^+^ vector, p15A ori, spec^r^	Derived from pYA3332 [26]
pQK163	Insertion of *crp* into pQK663	This work
pET-32a-*crp*	For the expression of *P. multocida* Crp	This work
pMC-Express	A broad host-range shuttle vector derived from pMIDG100, chloramphenicol^r^	[27]
pYA4278	pRE112 derivative, sacB mobRP4 R6K ori Cm^+^	[29]
pQK174	pYA4278-Δ*crp*	This work
pQK175	pYA4278-Δ*crp*::*kan*, for deletion of *crp* in *P. multocida* 0818	This work
pQK176	Insertion of *crp* into pMC-Express	This work
Strains		
S184	*S.* Typhimurium Δ*asd66*	Lab collection
S411	*S.* Typhimurium Δ*asd66* Δ*crp89*	Lab collection
*P. multocida* 0818	Wild-type and virulent. Capsular serotype A:1.	Lab collection
S416	*P.* multocida 0818 Δ*crp*::*kanR*	This work
χ7232	*E.coli* K-12, *endA1 hsdR17* (r_K_-, m_K_ *+*) *glnV44 thi-1 recA1 gyrA relA1* Δ(*lacZYA-argF*)*U169 λpir deoR* (ϕ*80dlac* Δ(*lacZ*)*M15*)	[30]
χ7213	*E.coli* K-12, *thi-1 thr-1 leuB6 glnV44 fhuA21 lacY1 recA1 RP4-2-Tc*::Mu λ*pir* ∆*asdA4* ∆*zhf-2*::*Tn10*	[30]

### Molecular and genetic procedures

Restriction digests and ligations were performed using enzymes purchased from New England Biolabs (NEB, Beverley, MA, USA) and TAKARA (Takara Bio Inc., Shiga, Japan), respectively, according to the manufacturer’s instructions. Plasmid DNA was extracted from bacteria using the TIANprep Mini Plasmid Kit (Tiangen Biotech Co., Ltd., Beijing, China), whereas genomic DNA was prepared using the cetyltrimethylammonium bromide method [31]. The DNA was amplified via PCR using PrimeSTAR Max DNA polymerase (Takara Bio Inc., Shiga, Japan) or *Taq* DNA polymerase (Tiangen Biotech) and purified using a DNA Purification Kit (Tiangen Biotech). The primers employed in this study were designed according to the published genome sequence of *P. multocida* strain Pm70 (GenBank, AE004439.1) [32] and are listed in Table 2. The DNA sequences were commercially determined by BGI Tech (BGI Tech Solutions Co., Ltd., Shenzhen, China), and sequence alignments were constructed using the Basic Local Alignment Search Tool (BLAST).

**Table 2:** Primers used in this work

Primer name	Sequence 5’-3’
C*crp*-F1	GCATGCCATGGTGCAAGAACAAATGCAAAC
C*crp*-R1	CGCGGATCCATGGATCGCATTTTAGCAGAG
C*crp*-F2	CGCGGATCCGTGCAAGAACAAATGCAAAC
C*crp*-R2	ATAAGAATGCGGCCGCATTTTAGCAGAGAACCGGG
pET-*crp*-F	GGGGTACCCAAGAACAAATGCAAACTAC
pET-*crp*-R	TGGATCCTTAGTGGTGGTGGTGGTGGTGTCTTGTACCGTAAACGACAATG
D*crp*-1 F	CGCATCTGGTGAACCTGTGT
D*crp*-1R	TACCTGCAGGATGCGGCCGCGGAAGACCTCCATAAACTAAT
D*crp*-2 F	CGCGGCCGCATCCTGCAGGTAATCCCCGGTTCTCTGCTAA
D*crp*-2R	GGCACGTTGCACATGAATC
*kan*-F	ATAAGAATGCGGCCGCTCAGTGGAACGAAAACTC
*kan*-R	CCTGCAGGTTAGAAAAACTCATCGAGCATC
Primer 1	AGGTGAAAAAGCCGAGACGC
Primer 2	GCGAACATCCCACCATTTGC
Primer 3	TGTTTGAAGCCTTGATTGAT
Primer 4	CTGATTCAGGTGAAAATATTG
Primer 5	CAATATTTTCACCTGAATCAG
Primer 6	GTCATTTCACCTGAATAAGC

### Plasmids and mutant strain construction

BLAST was applied to search for potential *P. multocida crp* gene (PM1157) via alignment of the amino acid sequences of *S.* Typhimurium Crp (Protein ID, NP_462369.1) and the genome of *P. multocida* Pm70. To clarify the potential gene in *P. multocida*, the PM1157 gene sequence was amplified from the *P. multocida* 0818 strain with the primers C*crp*-F1/C*crp*-R1. The amplified DNA fragment was inserted into pQK663 derived from pYA3332 [33] between the *Nco*I and *BamH*I digestion sites to generate pQK163, which was then transformed into the *S.* Typhimurium Δ*asd* Δ*crp* strain for a maltose fermentation assay. For expression of the Crp protein, the complete *P. multocida crp* sequence was amplified from *P. multocida* 0818 chromosomal DNA using the primers pET-*crp*-F and pET-*crp*-R. The PCR fragment was then purified and digested with *kpn*I*-*HF and *BamH*I*-*HF (NEB) and subsequently ligated to the pET-32a expression vector (Novagen Inc., Madison, WI, USA) between the *kpn*I and *BamH*I sites to generate pET-32a-*crp*. To complement the *crp* mutant in *P. multocida*, the complete *crp* gene was amplified from *P. multocida* 0818 genomic DNA using the primers C*crp*-F2/C*crp*-R2, and the amplified fragment was then digested and inserted into the *Not*I and *BamH*I sites of a shuttle vector pMC-Express [34] (kindly donated by Paul R Langford from Imperial College London) to generate pQK176. The plasmids pQK163 and pQK176 were transformed into the *crp* mutant strains S411 (*S.* Typhimurium Δ*asd* Δ*crp*) and S416 (*P. multocida* Δ*crp*), respectively, generating the corresponding complementary strains S411 (pQK163) and S416 (pQK176).

The *P. multocida* Δ*crp* mutant was constructed by allelic exchange using the suicide T-vector pYA4278 [35] as previously described [36]. Briefly, a 410-bp upstream segment and a 416-bp downstream segment of the *crp* gene were amplified with the primers D*crp*-1 F/D*crp*-1R and D*crp*-2 F/D*crp*-2R, respectively. The two segments were then linked via PCR using the primers D*crp*-1 F/D*crp*-2R. The PCR product was then ligated to *Ahd*I-digested pYA4278 to generate the plasmid pQK174. Next, the kanamycin resistance (*kanR*) cassette amplified from pYA4372 with the primers *kan*-F/*kan*-R was inserted into the *Not*I and *Sbf*I sites of pQK174 to generate the plasmid pQK175. This plasmid was subsequently introduced into *P. multocida* 0818 from *E. coli* χ7213 [37] via conjugation, and the Δ*crp* mutant designated S416 was selected on BHI agar containing kanamycin. Subsequently, the candidate mutant clones were verified by PCR screening using primers 1, 2, 3, 4, 5 and 6, which were designed based on the genomic sequence and the *kanR* cassette, as depicted in Fig. 2A. As a positive control, the 16S ribosomal RNA gene was amplified with the primers 16sRNA-F/16sRNA-R. Moreover, Crp expression was also measured in *P. multocida* 0818, S416 (*P. multocida* Δ*crp*) and S416 (pQK176) via western blotting with a 1:160-diluted polyclonal rabbit anti-Crp antibody.

### Purification of Crp protein and preparation of a polyclonal rabbit anti-Crp antibody

The plasmid pET-32a-*crp* was transformed to *E. coli* BL21 (DE3) cells (Tiangen Biotech) with ampicillin selection. Recombinants were harvested after 6 h of induction with 1 mM IPTG (isopropyl b-D-1-Thiogalactopyranoside). The Crp protein was purified using 6× His/Ni-NTA affinity chromatography. To prepare the polyclonal anti-Crp antibody, two female New Zealand white rabbits were subcutaneously immunized with Crp protein (1 mg) adjuvanted with Freund’s complete/incomplete adjuvant (Sigma-Aldrich, St. Louis, MO, USA) four times at 14-day intervals. The titers of the antisera were then analyzed using the immune agar diffusion test. When the titers of the two rabbit antisera reached at least 1:32, blood samples were collected within approximately 14 days after the last immunization to obtain the anti-Crp antibody.

### Maltose utilization test

To detect maltose utilization, S184 (*S*. Typhimurium Δ*asd*) harboring the control plasmid pQK663, S411 (S184 Δ*crp*) harboring pQK663 and S411 harboring the complementary plasmid pQK163 were grown at 37 °C for 18 h on MacConkey indicator plates containing 1 % maltose, and the colony color was observed [38].

### Phenotype determination

The growth curve of *P. multocida* strains was examined by recording their OD_600_ values every 2 h over a period of 14 h. The OMPs and LPS of *P. multocida* were extracted as previously described [36]. The OMP concentration was detected using a BCA Protein Assay Kit (Thermo Scientific, Rockford, IL, USA). The protein samples were subsequently diluted in sample buffer [50 mM Tris, 20 % glycerol, 4 % sodium dodecyl sulfate (SDS), 0.005 % bromophenol blue, and 5 % β-mercaptoethanol] and boiled for 5 min at 95 °C. The samples were then subjected to 12.5 % SDS-polyacrylamide gel electrophoresis (PAGE) followed by Coomassie Brilliant Blue R-250 staining (Sigma-Aldrich). Additionally, 10 μl of the LPS supernatant was diluted 1:10 in loading buffer, and the mixture was then treated with 1 μl of proteinase K (20 mg/ml, Sigma-Aldrich) at room temperature for 1 h and analyzed by 12.5 % SDS-PAGE followed by silver staining [30].

### Serum bactericidal assay

Duck serum was collected from healthy ducks and heat-inactivated via incubation for 30 min at 56 °C. The serum bactericidal activity against the *P. multocida* strains were then measured as previously described [39]. Briefly, bacteria were cultured overnight in BHI media to an OD_600_ of 0.8 to 0.9 at 37 °C and 180 rpm. The bacteria were re-suspended in phosphate-buffered saline (PBS) and diluted to a final concentration of 10^4^ CFU/ml. Aliquots (100 μl) of the bacterial suspensions were added to 900 μl of duck serum or heat-inactivated duck serum and incubated for 3 h at 37 °C with shaking. After incubation, the samples were placed on ice to inhibit further bacteriolysis. Serial dilutions of the samples in PBS were cultured on BHI agar plates and incubated at 37 °C overnight. The growth rate was calculated as the CFU per ml at 3 h divided by the CFU per ml at 0 h. All tests were performed in triplicate.

### Determination of the 50 % lethal dose (LD_50_) in ducks

All animal experiments in this study were conducted in strict accordance with the recommendations of the Guide for the Care and Use of Laboratory Animals of the Ministry of Science and Technology of China. All animal procedures were approved by the Animal Care and Use Committee of Sichuan Agricultural University (No. XF2014-18).

To determine the LD_50_ of *P. multocida* strains, duck infection studies were conducted as described previously [36]. Overnight cultures of *P. multocida* strains in BHI medium were diluted 1:100 in fresh medium and further cultured at 37 °C with shaking to an OD_600_ of 0.8–0.9. The numbers of viable bacteria were then counted, and the bacteria were diluted in 100 μl of PBS to obtain cultures of 10^2^ to 10^10^ CFU/ml. Various doses of *P. multocida* 0818 or S416 (Δ*crp*) were then intranasally inoculated into groups of 2-week-old Sheldrake ducks. The clinical symptoms and health of the ducks were monitored over a period of 2 weeks after infection. The LD_50_ was calculated following the method described by Reed and Muench, and the experiment was repeated twice.

### RNA extraction and sequencing

For preparation of bacterial RNA samples, *P. multocida* 0818 and S416 (Δ*crp*) were grown in BHI medium in triplicate with shaking. Once the OD_600_ of the cultures reached 0.8, the bacteria were harvested, and the total RNA from each sample was extracted and purified using the TRIzol reagent (Invitrogen, CA, USA). Contaminating DNA was removed from the total RNA samples with DNase I (NEB Inc., USA) at 37 °C for 10 min, and ribosomal RNA was eliminated using a MICROBExpress kit (Thermo Fisher Scientific Inc., CA, USA). The RNA quality and concentration were determined using a Nanodrop spectrophotometer (Thermo Fisher Scientific Inc., USA). A cDNA library was then constructed using a TruSeq RNA Sample Preparation Kit (Illumina, San Diego, CA, USA), and the Illumina Hiseq2500/MiSeq platform (Illumina) was used for RNA deep sequencing, which was conducted at Majorbio Bio-pharm Biotechnology Co., Ltd. (Shanghai, China).

Sequence analysis was performed as previously described [40]. In brief, clean reads were obtained from the sequenced raw data using FASTQC and NGS QC TOOLKIT and *de novo* assembled by Trinity software. The TGICL package was then applied to generate valid unigenes, and the Bowtie 2 and eXpress software programs were used for the mapping of the clean reads from each sample to unigenes based on the reference genome sequence of *P. multocida* Pm70. The number of mapped reads relative to each gene was measured using the RPKM method. The RSEM and edgeR software packages were subsequently applied for the screening of unigene transcripts with differential expression between *P. multocida* 0818 and S416.

### Immunization and challenge

One-week-old Sheldrake ducks were intranasally immunized with 1 × 10^3^ CFU of S416 or PBS twice at an interval of 10 days. The S416-immunized group included 16 ducks, and the PBS control group included 14 ducks. Serum and bile were collected from six randomly selected ducks from both groups on Day -3 and Day 20, respectively, and stored at -80 °C until analysis. For the challenge assay, the ducks in the S416-immunized and PBS groups were intranasally inoculated with a lethal dose of *P. multocida* strain 0818, approximately 1 × 10^7^ CFU. The health status of the animals was monitored and recorded every day for two weeks post-challenge, and the deceased ducks were routinely subjected to bacterial isolation.

### Enzyme-linked immunosorbent assay (ELISA)

The serum IgY and bile IgA levels were detected via indirect ELISA as previously described [36]. A 96-well ELISA microplate was coated with 1 × 10^10^ CFU heating-inactivated *P. multocida* 0818 or 0.25 μg/ml purified OMPs and incubated at 4 °C overnight. After three washes with PBST, the plate was then blocked with 2 % BSA in PBS. The serum samples were diluted 1:100 in PBS containing 1 % BSA, and the bile samples were diluted 1:40. After the plate was washed again, 100 μl of these dilutions was added to each well. After 1 h of incubation at 37 °C, the plate was incubated with 100 μl of 1:5000-diluted alkaline phosphatase (AP)-labeled mouse anti-duck IgY or IgA (AbD Serotec, Puchheim, Germany) at 37 °C for 1 h. AP solution (Sigma-Aldrich) was added for coloration, and the reaction was terminated by the addition of 100 μl of 0.2 M NaOH. The optical density (OD) value at 415 nm was measured using a microplate reader (Bio-Rad Laboratories, Richmond, CA, USA).

### Statistical analyses

The GraphPad Prism 5 software package (Graph Software, San Diego, CA, USA) was used for the statistical analyses. The data are expressed as the means ± standard deviations (SD) and were evaluated using Student’s *t* test with significance levels set to 0.05 and 0.01. The animal experiments were performed at least twice, and the in vitro experiments were conducted independently three times in triplicate.

## Results

### Cloning and characterization of the *crp* gene of *P. multocida*

**Fig. 1:**
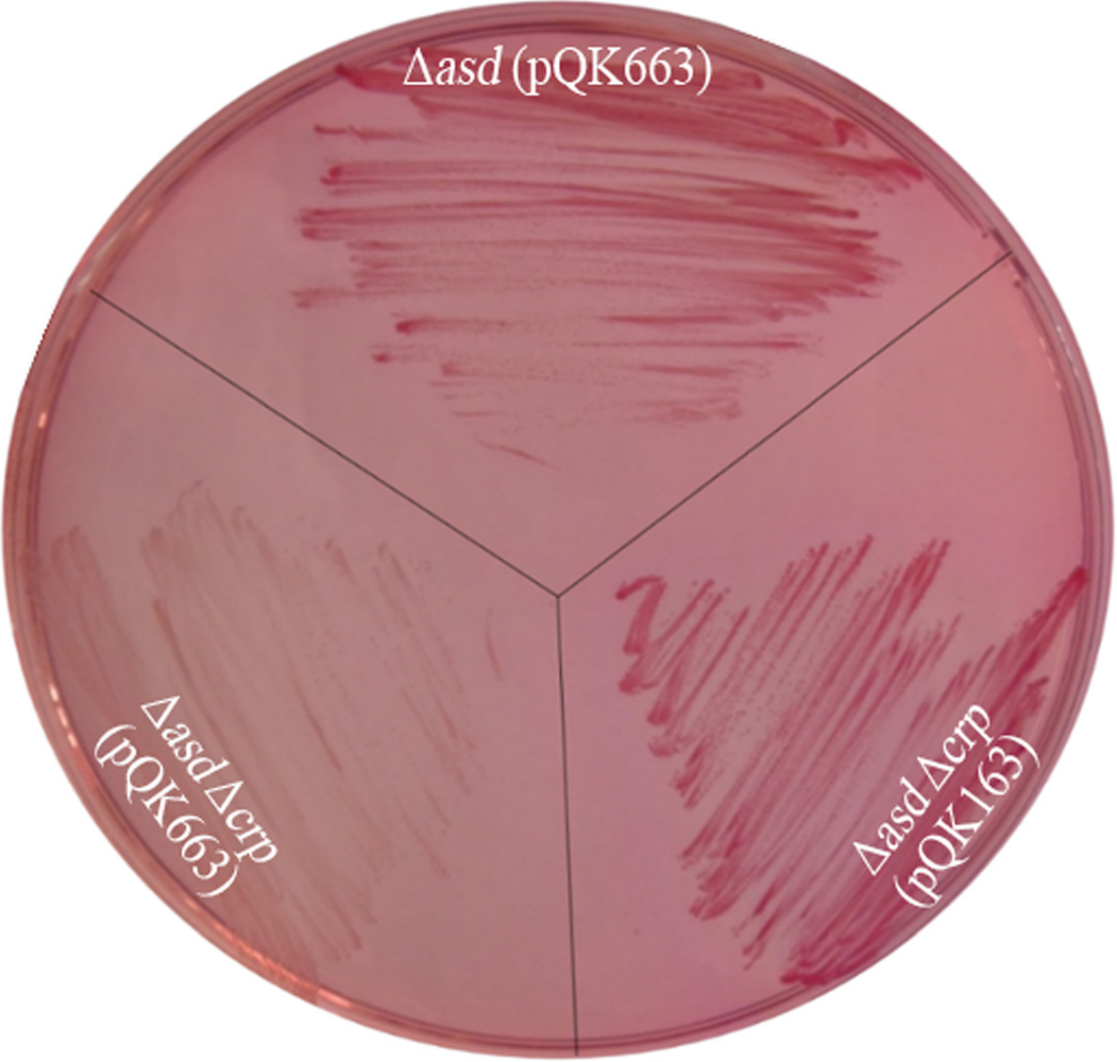
Detection of maltose fermentation in *S*. Typhimurium. Three *S.* Typhimurium strains, S184 (Δ*asd*) harboring the control plasmid pQK663, S411 (Δ*asd*Δ*crp*) harboring pQK663 and S411 (Δ*asd*Δ*crp*) harboring the complementary plasmid pQK163 (PM1157), were cultured on MacConkey maltose agar to observe the colors of the clones

The coding region of the suspected *crp* gene (PM1157) in *P. multocida* strain 0818 was cloned via PCR, then sequenced and deposited in GenBank (accession number, KU507499). The PM1157 sequence was 630 bp in length and shared 73 % nucleotide identity with both *S*. Typhimurium *crp* (Gene ID, 1254989) and *E. coli crp* (Gene ID, 947867) over 604 nucleotides. Additionally, the 630 bp sequence of the *P. multocida* crp gene was predicted to encode a 209-amino acid polypeptide, which showed 86 and 87 % identity to *S*. Typhimurium Crp (Protein ID, NP_462369.1) and *E. coli* Crp (Protein ID, NP_417816.1) over 205 amino acids, respectively. The deletion of *crp* resulted in defects in maltose fermentation in *S*. Typhimurium [38]. To clarify the nature of the cloned sequence, we evaluated whether the PM1157 gene could restore maltose fermentation in *S.* Typhimurium *crp* mutant. Maltose fermentation was detected in S184 (*S*. Typhimurium Δ*asd*) harboring pQK663 (control plasmid), S411 (*S*. Typhimurium Δ*asd* Δ*crp*) harboring pQK663 and S411 harboring pQK163 (pQK663-PM1157). As shown in Fig. 1, S184 (pQK663) and S411 (pQK163) produced red clones, whereas S411 (pQK663) produced white clones on MacConkey maltose agar. Thus, the suspected *crp* gene (PM1157) complemented the *Salmonella* Δ*crp* mutant, allowing the utilization of maltose.

### Construction of the non-polar Δ*crp* mutant in *P. multocida* 0818

**Fig. 2:**
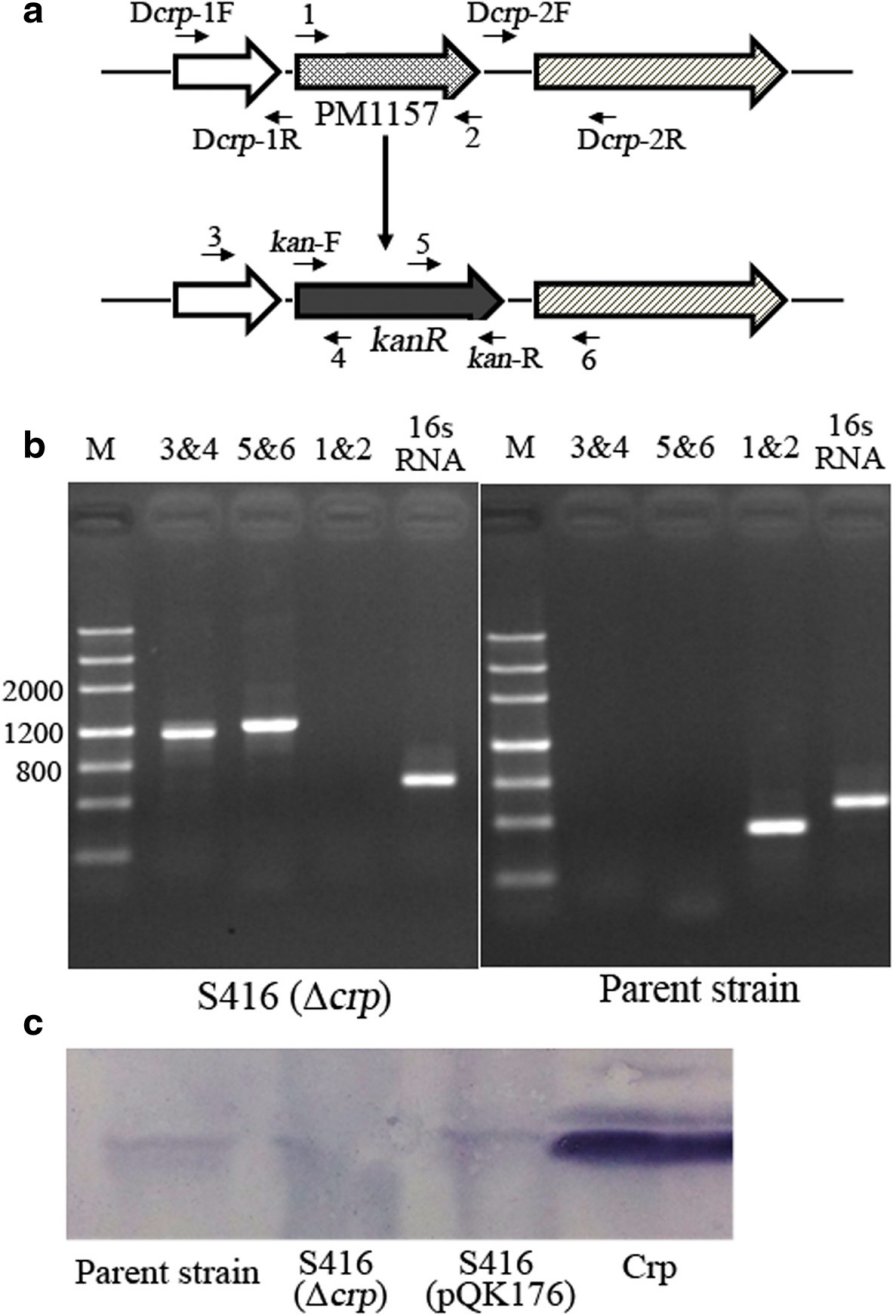
Construction of the Δ*crp* mutant in the *P. multocida* 0818 strain. **a** Schematic of the strategy used for deletion of the target *crp* gene (PM1157). The *P. multocida crp* gene was replaced with a *kanR* cassette via homologous recombination. Three pairs of primers, 1& 2, 3& 4, and 5& 6, were designed to select and characterize the mutant clones. **b** Characterization of the constructed Δ*crp* mutant via PCR. The parent strain and Δ*crp* mutant were identified using the primers 1&2, 3&4 and 5&6. M refers to the DNA marker; 16sRNA indicates amplification of the positive gene in both strains. **c** Detection of Crp expression in *P. multocida* stains. The parent strain, S416 (Δ*crp*) and S416 (pQK176) were grown in BHI media and collected at an OD_600_ of 1.0, and the expression of Crp was then measured in these strains with an anti-Crp antibody via western blotting. Crp refers to purified protein and served as a positive control

To determine the role of *crp* (PM1157) in *P. multocida*, the Δ*crp* mutant S416 was constructed via suicide plasmid-mediated homologous recombination and characterized through PCR using three pairs of primers, 1&2, 3&4, and 5&6 (Fig. 2A). The DNA segment containing the PM1157 upstream sequence and a partial *kanR* cassette (3&4) and the DNA segment containing the PM1157 downstream sequence and a partial *kanR* cassette (5&6) were present in the S416 strain but not in the parent strain (*P. multocida* 0818), whereas the complete PM1157 sequence (1&2) was only present in the parent strain (Fig. 2B). The positive control 16S RNA could be amplified from both strains. Crp expression was also measured using a polyclonal anti-Crp antibody. Crp was expressed in the parent strain and the complementary strain S416 (pQK176), but not in S416 (Fig. 2C), demonstrating that the *crp* gene was successfully deleted in S416. Moreover, the *crp* mutation was stable for more than 20 passages (data not shown).

### Phenotype characterization of the *P. multocida crp* mutant

**Fig. 3:**
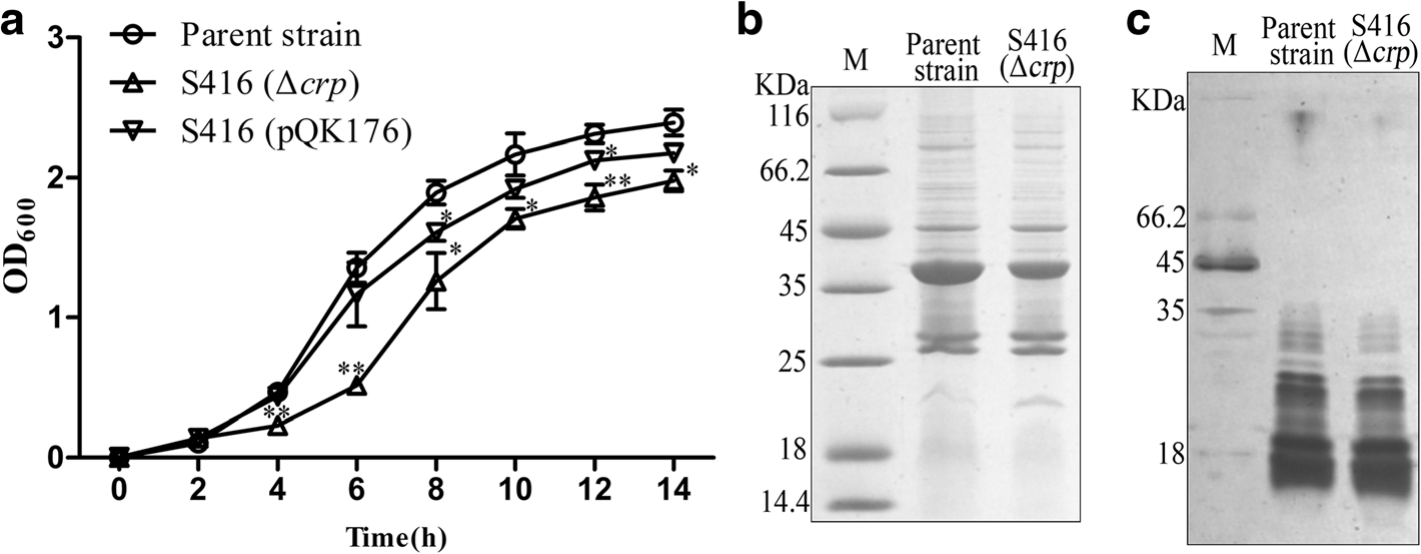
Phenotype detection of the *P. multocida* Δ*crp* mutant. **a** Analysis of the growth curve. The parent strain, S416 (Δ*crp*) and S416 (pQK176) were grown in BHI broth or BHI broth supplemented with kanamycin, and the OD_600_ values were measured every 2 h over a period of 14 h. The data are expressed as the means ± SD, and the asterisks indicate significant differences compared with the parent strain. **b** and **c** Profiles of the OMPs and LPS of *P. multocida*. OMPs or LPSs were isolated from the parent strain or the Δ*crp* mutant and then analyzed by SDS-PAGE. Coomassie blue staining and silver staining were then performed to visualize the OMPs (**b**) and LPSs (**c**), respectively. M refers to the protein marker

To detect the influence of *crp* deletion, the growth curve, OMP and LPS profiles, and serum complement sensitivity were evaluated in the parent strain (*P. multocida* 0818), S416 (*P. multocida* Δ*crp*) and S416 (pQK176). The parent strain showed a typical growth curve, with a short lag phase (0–2 h), followed by a log phase during which major bacterial growth occurred (2–10 h) and then a stationary phase (10–14 h; Fig. 3A). In contrast, S416 grew more slowly after 2 h, particularly between 4 and 8 h. During this phase, the OD values of S416 were significantly lower than those of the parent strain (Fig. 3A). The complementary strain, S416, which harbors pQK176, partially restored the defective growth (Fig. 3A), but the OD values recorded at 8 h and 12 h remained lower than those of the parent strain.

The S416 strain displayed OMP and LPS profiles similar to those of the parent strain (Figs. 3B and C). Specifically, these profiles primarily consisted of proteins larger than 25 kDa and short-length LPS, respectively. Moreover, both the parent strain and S416 (pQK176) grew rapidly in either untreated or heat-treated duck serum, whereas S416 (Δ*crp*) was rapidly killed in untreated serum but grew in heat-treated serum (Table 3). The growth rates of S416 significantly differed between untreated and heat-treated serum (Table 3), demonstrating that the deletion of *crp* increased sensitivity to complement-mediated killing.

**Table 3:** Serum bactericidal assay

Strains	Serum heat treatment	Growth rate^a^
Parent strain	-	16.4 ± 1.2
	+	16.6 ± 0.8
S416 (Δ*crp*)	-	3.0 ± 0.3^b^
	+	10.7 ± 0.4
S416 (pQK176)	-	11.1 ± 0.8
	+	12.1 ± 1.0

^a^, The data are means and SD of three independent experiments

^b^, The difference in sensitivity between S416 in heated or unheated serum was determined to be very significant (*p* < 0.01)

### Determination of the virulence of wild-type *P. multocida* and the Δ*crp* mutant

To determine the effects of the *crp* mutation on bacterial virulence, the LD_50_ values of the parent strain and S416 (*P. multocida* Δ*crp*) were assessed in a duck animal model. The results showed that the LD_50_ of S416 was 7.4 × 10^6^ CFU, which was 85-fold higher than that of the parent strain, whose LD_50_ was 8.66 × 10^4^ (Table 4).

**Table 4:** Determination of the LD_50_ of *P. multocida* 0818 and the Δ*crp* mutant

Route	Strains	Challenge dose (CFU) and survival							LD_50_ (CFU)
		10^3^	10^4^	10^5^	10^6^	10^7^	10^8^	10^9^	
Intranasal	*P. multocida* 0818	5/5	7/8	8/16	1/16	0/16	0/8	0/8	8.66 × 10^4^
	S416 (Δ*crp*)	-	8/8	7/8	11/16	7/16	1/16	0/8	7.4 × 10^6^

-, Not detected

### Identification of genes regulated by *crp* in *P. multocida*

Transcriptome sequencing was performed to screen for genes regulated by *crp* in *P. multocida*. Compared with the parent strain, 186 genes in addition to *crp* itself were differentially expressed in the S416 (Δ*crp*) strain during the exponential growth stage. Specifically, 92 of these genes were up-regulated, and 94 genes were down-regulated (see Additional file 1). Genes exhibiting fold-differences in transcription greater than 3.5 between the parent strain and the Δ*crp* mutant are listed in Table 5. A KEGG enrichment analysis showed that the regulated genes were significantly involved in six pathways, including two-component systems, arginine and proline metabolism, pyruvate metabolism, nitrogen metabolism, and oxidative phosphorylation (see Additional file 2).

**Table 5:** A partial list of differentially expressed genes between the parent strain and the Δ*crp* mutant

Gene ID^a^	Name	Description	Fold Change (log2)
Genes down-regulated in the Δ*crp* strain			
1244122	*fcbD*	acetylgalactosaminyl-proteoglycan 3-beta-glucuronosyltransferase	2.55
1244154	*potE*	putrescine:ornithine antiporter	2.44
1244150	*PM0803*	TonB-dependent receptor	2.32
1244125	*wza*	sugar ABC transporter substrate-binding protein	2.19
1243664	*iscR*	Rrf2 family transcriptional regulator	2.07
1244127	*PM0780*	sugar ABC transporter permease	1.93
1243665	*PM0318*	cysteine desulfurase	1.87
1244088	*PM0741*	ligand-gated channel protein	1.85
1244123	*ugd*	UDP-glucose 6-dehydrogenase	1.85
1244252	*miaA*	tRNA delta(2)-isopentenylpyrophosphate transferase	1.82
1243649	*PM0302*	sodium:proton antiporter	1.81
Genes up-regulated in the Δ*crp* strain			
1244768	*oadG*	oxaloacetate decarboxylase subunit gamma	3.19
1243370	*nrfA*	cytochrome C nitrite reductase subunit c552	3.13
1244503	*PM1156*	hypothetical protein	2.64
1244939	*napF*	ferredoxin	2.62
1243678	*ompW*	membrane protein	2.55
1243988	*bioD*	dithiobiotin synthetase	2.51
1243371	*nrfB*	cysteine dioxygenase	2.46
1243373	*nrfD*	formate-dependent nitrite reductase subunit NrfD	2.33
1243600	*PM0253*	hypothetical protein	2.25
1243934	*PM0587*	hypothetical protein	2.23
1244940	*napD*	nitrate reductase	2.23
1243763	*PM0416*	glucose-6-phosphate isomerase	2.11
1243372	*nrfC*	formate-dependent nitrite reductase subunit NrfC	2.09
1243771	*PM0424*	hypothetical protein	2.00
1243606	*PM0259*	cytidine deaminase	2.00
1244726	*PM1379*	D-ribose transporter ATP binding protein	1.98
1244035	*PM0688*	membrane protein	1.95
1243893	*ppc*	phosphoenolpyruvate carboxylase	1.92
1245036	*tatA*	preprotein translocase subunit TatA	1.89
1244941	*napA*	nitrate reductase catalytic subunit	1.83
1244769	*oadA*	oxaloacetate decarboxylase	1.82

### Evaluation of immune responses and the protection rate conferred by the Δ*crp* mutant

**Fig. 4:**
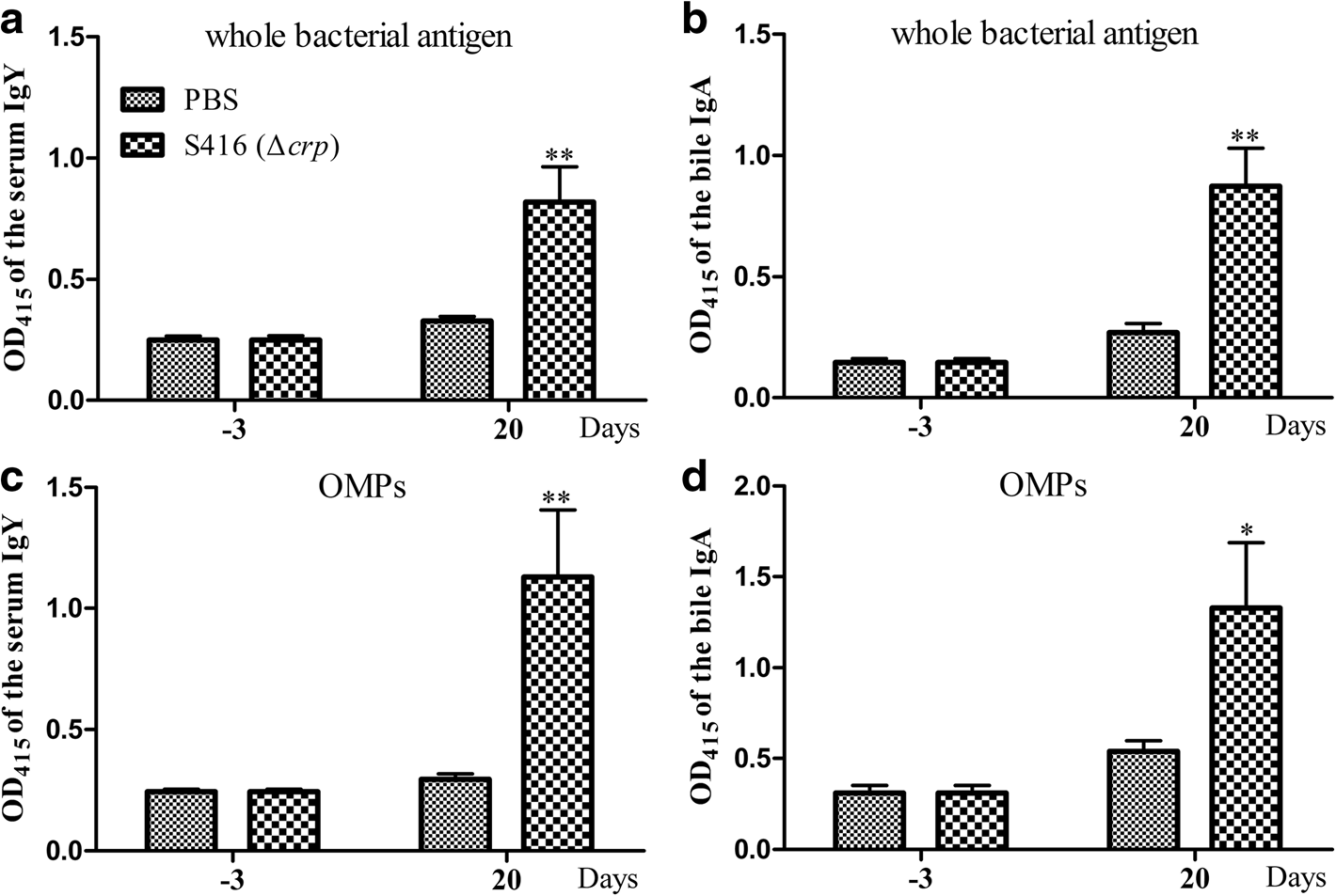
Antibody responses induced by the Δ*crp* mutant in ducks. Ducks were intranasally immunized twice with the Δ*crp* mutant at an interval of 10 days. The serum IgY responses specific to whole bacterial antigen (**a**) and OMPs (**b**) and the bile IgA responses specific to whole bacterial antigen (**c**) and OMPs (**d**) were then assessed 3 days before immunization and 20 days post-immunization via indirect ELISA. The data are expressed as the means ± SD and were analyzed at significance levels of 0.05 (*) and 0.01 (**)

The antibody responses induced by S416 (Δ*crp*) were detected by ELISA post-immunization. As shown in Fig. 4, no specific serum IgY or bile IgA against bacteria antigen were detected in both the S416-immunized group and the PBS group three days prior to immunization. In contrast to the PBS group, S416-immunized group induced significantly higher levels of serum IgY to whole bacteria antigen and OMPs 20 days post-immunization (Figs. 4A and B). In addition, the bile IgA levels against whole bacteria antigen and OMPs were significantly increased in the S416-immunized group compared with the PBS group 20 days post-immunization (Figs. 4C and D). Moreover, 60 % of the ducks in the immunized group survived and steadily gained weight after challenge, whereas all control ducks were dead within one week (Table 6). Thus, the Δ*crp* mutant induced 60 % protection against challenge with a dosage of 100-fold of the LD_50_ of wild-type *P. multocida* 0818 in ducks.

**Table 6:** Survival rate conferred by the Δ*crp* mutant

Group	Immunization	Challenge	Survival	Protection rate
Immune group	10^3^ CFU S416 (Δ*crp*)	10^7^ CFU *P. multocida* 0818	6/10	60 %
Control	PBS	10^7^ CFU *P. multocida* 0818	0/8	0

## Discussion

Global regulators play a vital role in the adaptation of bacteria to the environmental changes that are encountered during infection. Despite the broad spectrum of diseases caused by *P. multocida* and their worldwide economic impact, only a few regulators have been characterized in *P. multocida*, including Fis (nucleoid-associated proteins) [41], Fur (ferric uptake regulation) [42], PhoP [36] and FnrP [43]. These regulators are all associated with bacterial virulence. Here, we first identified the homologue of the *crp* gene in *P. multocida* and showed that PM1157 is the functional *crp* gene. Two pieces of evidence support this conclusion: 1) PM1157 exhibited a high degree of amino acid identity (86 %) with corresponding proteins from other bacteria, including *E. coli* and *Salmonella enterica*; and 2) the PM1157 gene of *P. multocida* was able to restore maltose fermentation in the *Salmonella crp* mutant. Thus, gene PM1157 of *P. multocida* is a *crp* gene that is interchangeable with *Salmonella crp*.

The resistance of S416 (*P. multocida* Δ*crp*) to duck serum complement was significantly reduced compared with that of the parent stain. Previous reports have demonstrated that the long O-antigen chain of *Salmonella* serovars or the presence of certain outer membrane proteins of some bacteria, such as *Salmonella* Rck and *Haemophilus influenzae* P5, contributes to complement resistance [44–46]. Capsular polysaccharide also prevents the complement-mediated clearance of *Salmonella enterica* serotype Typhi [47] and *P. multocida* [48]. Here, we showed that the LPS profile of S416 was similar to that of the parent strain, mainly containing short oligosaccharides. Several OMP genes, rather than capsule synthesis genes, were differentially expressed in the absence of the *crp* gene (see Additional file 1). Therefore, we speculated that some of these OMPs were responsible for the decrease in complement resistance, which should be confirmed in a later study. In addition, the virulence of the S416 strain decreased 85-fold after intranasal inoculation, and this reduction was much less pronounced in the *P. multocida* Δ*crp* mutant than in the *Salmonella* Δ*crp* mutant, for which virulence was reduced by five orders of magnitude [49]. Similar results have been observed for a *crp* mutation in *Edwardsiella ictaluri* [50], indicating that *crp* does not necessarily impact virulence, or that its impact on virulence is species-specific, or that other genes in *P. multocida* may compensate for a loss of *crp* to minimize adverse effects.

Crp–cAMP can directly control a minimum of 378 promoters and perhaps > 500 genes in *E. coli* [22]. In this study, *crp* was shown to influence the transcription of 186 genes, including 92 up-regulated genes and 94 down-regulated genes in *P. multocida* (see Additional file 1). This finding suggested that *crp* acts as both a positive and negative regulator. The majority of differentially expressed genes participate in metabolism, including carbon metabolism, arginine and proline metabolism, pyruvate metabolism, nitrogen metabolism and ABC transporters. Therefore, Crp plays an important role in the metabolism of *P. multocida*. The deletion of the *crp* gene also resulted in the down-regulation of two global regulators (*fnr* and *iscR*) and up-regulation of seven regulators (*pgtC*, *qseB*, *arcA*, *ttrC*, *ttrB*, *rraA* and *isrR*). Among these regulators, *fnr*, *qseB* and *arcA* have been shown to positively regulate the virulence of pathogenic bacteria. Deletion of *fnr*, *qseB* or *arcA* significantly reduces the virulence of *S.* Typhimurium [51], *Aeromonas hydrophila* [52] and *Vibrio cholerae* [53], respectively. Thus, the down-regulation of *fnr* observed in the *P. multocida* Δ*crp* mutant may partially account for the decreased virulence of this strain, and the up-regulation of *qseB* and *arcA* may have attenuated this decrease.

Vaccines are the most economical and effective means to control infectious disease. Compared with vaccines based on a subunit or dead bacteria, attenuated live vaccines are advantageous because they can induce long-term immunity and confer good protection [4]. A significant number of attenuated live *P. multocida* vaccines have been successfully developed by targeting capsule genes [54, 55], toxin genes [56] and *aroA* [57], which elicit protective responses in mice, livestock or poultry. Here, we evaluated the vaccine potential of the *P. multocida* Δ*crp* mutant. Because circumstantial evidence has implicated the respiratory tract as the main route of *P. multocida* infection, we selected the intranasal route to immunize ducks. The *P. multocida* Δ*crp* mutant induced potent serum IgY and bile IgA responses in ducks, indicating the high immunogenicity of the Δ*crp* mutant. Additionally, this mutant provided 60 % protection against challenge with the *P. multocida* virulent strain at a dosage of 100-fold of the LD_50_ (Table 6). This level of protection was lower than that conferred by the *Salmonella crp* mutant [13]. The immunization dose of an attenuated *P. multocida* strain has been demonstrated to be related to the level of protection against challenge [58]; animals receiving a higher vaccine dose (10^9^ or 10^8^ CFU of the attenuated strain) are less affected clinically, bacteriologically, and pathologically through wild-type challenge compared with the administration of a lower dose of 10^7^ CFU [58]. Because the *P. multocida* Δ*crp* mutant was not fully attenuated, only low doses of this mutant (10^3^ CFU) were used to immunize the ducks. We speculate that the low immunization dose might have been responsible for the limited protection observed. However, other studies have also shown that fully attenuated bacterial strains such as the *Edwardsiella tarda aroA* mutant fail to provide effective protection against virulent challenge even at a high dose of immunization, probably because of reduced immunogenicity or loss of protective antigens [59–61]. Therefore, it is vital to retain or improve bacterial immunogenicity while achieving attenuation through the deletion of virulence regulators. Furthermore, bacteria with mutations in two or more regulator genes are better attenuated than those with single-gene mutations in some cases and provide high vaccine potency [62]. Thus, mutations in other regulator genes need to be selected and introduced into the *crp* mutant to achieve full attenuation for vaccine development in a later study.

## Conclusions

The PM1157 gene is the *crp* homologue of *P. multocida*. The deletion of the *crp* gene has an inhibitory effect on bacterial growth and bacterial resistance to serum complement, without affecting the electrophoretic bands of LPS and OMPs in vitro. Furthermore, the *P. multocida crp* mutant was attenuated and provided 60 % protection in ducks. The present study provides a basis that will allow the mechanisms of *crp*-regulated genes to be explored to ultimately develop a platform for an attenuated vaccine against *P. multocida*.

## Abbreviations

AP, alkaline phosphatase; BHI, brain heart infusion; DAP, diaminopimelic acid; *E. coli*, *Escherichia coli*; ELISA, enzyme-linked immunosorbent assay; *kanR*, kanamycin resistance; LB, Luria-Bertani; LD_50_, 50 % lethal dose; LPS, lipopolysaccharide; OMPs, outer membrane proteins; *P. multocida*, *Pasteurella multocida*; PAGE, polyacrylamide gel electrophoresis; PBS, phosphate-buffered saline; *S.* Typhimurium, *Salmonella enterica* serovar Typhimurium; SDS, sodium dodecyl sulfate

## Additional files

Additional file 1: Total differentially expressed genes between the parent strain and the Δ*crp* mutant. (PDF 142 kb) Additional file 2: KEGG enrichment analysis of the differentially expressed genes in the Δ*crp* mutant. KOBAS software was used to analyze the *crp*-regulated genes in KEGG pathways. Each column in A and B indicates one pathway, and the abscissa represents the name and classification of the pathway. The column color refers to the significance, and the depth of the color directly correlates with the degree of significance. *, *p* < 0.05; **, *p* < 0.01; ***, *p* < 0.001. (TIF 367 kb)
