# Supplementary material for: Identification of the crp gene in avian Pasteurella multocida and evaluation of the effects of crp deletion on its phenotype, virulence and immunogenicity
Source: BMC Microbiol. 2016 Jun 24;16:125. doi: 10.1186/s12866-016-0739-y (PMC4921010; doi:10.1186/s12866-016-0739-y)
Supplement: Additional file 1: — Total differentially expressed genes between the parent strain and the Δcrp mutant. (PDF 142 kb) [file 12866_2016_739_MOESM1_ESM.pdf]

Gene ID[a]	Name	Description	Fold Change (log2)
**Genes up-regulated in Δ*crp* strain**			
1244768	*oadG*	oxaloacetate decarboxylase subunit gamma	3.19
1243370	*nrfA*	cytochrome C nitrite reductase subunit c552	3.13
1244503	*PM1156*	hypothetical protein	2.64
1244939	*napF*	ferredoxin	2.62
1243678	*ompW*	membrane protein	2.55
1243988	*bioD*	dithiobiotin synthetase	2.51
1243371	*nrfB*	cysteine dioxygenase	2.46
1243373	*nrfD*	formate-dependent nitrite reductase subunit NrfD	2.33
1243600	*PM0253*	hypothetical protein	2.25
1243934	*PM0587*	hypothetical protein	2.23
1244940	*napD*	nitrate reductase	2.23
1243763	*PM0416*	glucose-6-phosphate isomerase	2.11
1243372	*nrfC*	formate-dependent nitrite reductase subunit NrfC	2.09
1243771	*PM0424*	hypothetical protein	2.00
1243606	*PM0259*	cytidine deaminase	2.00
1244726	*PM1379*	D-ribose transporter ATP binding protein	1.98
1244035	*PM0688*	membrane protein	1.95
1243893	*ppc*	phosphoenolpyruvate carboxylase	1.92
1245036	*tatA*	preprotein translocase subunit TatA	1.89
1244941	*napA*	nitrate reductase catalytic subunit	1.83
1244769	*oadA*	oxaloacetate decarboxylase	1.82
1243612	*pepT*	peptidase M20	1.80
1244805	*PM1458*	pesticidal protein Cry1Aa	1.78
1243723	*tbpA*	thiamine ABC transporter substrate-binding protein	1.78
1244770	*oadB*	oxaloacetate decarboxylase subunit beta	1.72
1244620	*lsrR*	transcriptional regulator	1.72
1244564	*PM1217*	TonB-dependent receptor protein	1.71
1244942	*napG*	quinol dehydrogenase	1.68
1244070	*ttrB*	tetrathionate reductase subunit B	1.65
1244014	*PM0667*	ferritin	1.60
1244633	*uspA*	universal stress protein A	1.58
1245218	*eno*	enolase	1.55
1243959	*PM0612*	membrane protein	1.54
1244945	*napC*	cytochrome C	1.50
1244943	*napH*	quinol dehydrogenase	1.47
1244721	*PM1374*	hypothetical protein	1.47
1243872	*PM0525*	arabinose 5-phosphate isomerase	1.44
1243352	*ccmA*	cytochrome C biogenesis protein CcmA	1.43
1243866	*PM0519*	hypothetical protein	1.42
1243713	*PM0366*	hypothetical protein	1.42
1245101	*dmsA*	dimethyl sulfoxide reductase subunit A	1.41

1244724	*rbsB*	hypothetical protein	1.39
1244021	*PM0674*	hypothetical protein	1.38
1244825	*PM1478*	hypothetical protein	1.36
1243621	*PM0274*	C4-dicarboxylate ABC transporter	1.36
1244647	*PM1300*	peptidase M20	1.35
1245037	*tatB*	preprotein translocase subunit TatB	1.35
1245025	*PM1678*	hypothetical protein	1.34
1245244	*PM1897*	hypothetical protein	1.30
1244170	*fumC*	fumarate hydratase	1.30
1243546	*frdC*	fumarate reductase	1.29
1245002	*PM1655*	hypothetical protein	1.28
1243896	*PM0549*	arginine repressor	1.27
1244146	*yciS*	membrane protein	1.27
1244278	*PM0931*	hypothetical protein	1.25
1243887	*malQ*	4-alpha-glucanotransferase	1.25
1244871	*PM1524*	cupin	1.25
1244646	*PM1299*	C4-dicarboxylate ABC transporter	1.24
1243545	*frdD*	fumarate reductase	1.23
1245252	*cah*	carbonic anhydrase	1.22
1244476	*arfA*	hypothetical protein	1.22
1243547	*frdB*	fumarate reductase iron-sulfur subunit	1.21
1243566	*arcA*	transcriptional regulator	1.18
1243871	*kdsC*	3-deoxy-D-manno-octulosonate 8-phosphate phosphatase	1.18
1244954	*ftsB*	cell division protein FtsB	1.17
1245165	*PM1818*	virulence factor	1.17
1245281	*PM1934*	hypothetical protein	1.17
1244845	*PM1498*	potassium transporter	1.17
1244515	*rraA*	ribonuclease activity regulator protein RraA	1.16
1243753	*fdoI*	formate dehydrogenase	1.16
1244604	*PM1257*	transcriptional regulator	1.15
1244903	*comF*	competence protein ComF	1.14
1245100	*PM1753*	membrane protein [Pasteurella hypothetical protein PM1753	1.14
1243563	*PM0216*	molybdenum-pterin-binding protein	1.14
1244846	*hemG*	protoporphyrinogen oxidase	1.13
1244714	*PM1367*	DeoR family transcriptional regulator	1.13
1244725	*rbsC*	ribose ABC transporter permease	1.11
1243972	*moaA*	molybdenum cofactor biosynthesis protein MoeA	1.10
1244662	*corA*	magnesium/nickel/cobalt transporter CorA	1.10
1244017	*PM0670*	hypothetical protein	1.09
1243349	*maeB*	malic enzyme	1.09
1243975	*PM0628*	integration host factor subunit alpha	1.09
1243561	*qseB(phoP)*	XRE family transcriptional regulator	1.07

1244022	*PM0675*	N-acetyl-D-glucosamine kinase	1.07
1244544	*PM1197*	VatB protein [Pasteurella VatB protein	1.06
1243583	*dppA*	peptide transporter	1.06
1244069	*PM0722*	hypothetical protein	1.05
1243773	*PM0426*	ABC transporter	1.03
1243358	*dsbE*	thiol:disulfide interchange protein	1.02
1245238	*PM1891*	hypothetical protein	1.01
1243354	*ccmC*	heme ABC transporter permease	1.01
1244334	*PM0987*	glyoxalase I	1.00
**Genes down-regulated in Δ*crp* strain**			
1244122	*fcbD*	acetylgalactosaminyl-proteoglycan 3-beta-glucuronosyltransferase	2.55
1244154	*potE*	putrescine transporter	2.44
1244150	*PM0803*	TonB-dependent receptor	2.32
1244125	*wza*	sugar ABC transporter substrate-binding protein	2.19
1243664	*iscR*	Rrf2 family transcriptional regulator	2.07
1244127	*PM0780*	sugar ABC transporter permease	1.93
1243665	*PM0318*	cysteine desulfurase	1.87
1244088	*PM0741*	ligand-gated channel protein	1.85
1244123	*ugd*	UDP-glucose 6-dehydrogenase	1.85
1244252	*miaA*	tRNA delta(2)-isopentenylpyrophosphate transferase	1.82
1243649	*PM0302*	sodium:proton antiporter	1.81
1244730	*ordL*	oxidoreductase	1.80
1245245	*PM1898*	peptidyl-prolyl cis-trans isomerase	1.78
1243635	*lldD*	L-lactate dehydrogenase	1.77
1244851	*PM1504*	dimethyladenosine transferase	1.76
1244459	*PM1112*	RNA helicase	1.70
1244124	*PM0777*	hypothetical protein	1.68
1244580	*pdxT*	glutamine amidotransferase	1.67
1243667	*iscA*	iron-sulfur cluster assembly protein	1.66
1244254	*hflX*	GTPase HflX	1.63
1244579	*pdxS*	pyridoxal biosynthesis protein	1.54
1243532	*PM0185*	6-carboxy-5,6,7,8-tetrahydropterin synthase	1.54
1244126	*kpsE*	capsule polysaccharide transporter	1.51
1244120	*kpsC*	capsule polysaccharide transporter	1.50
1244118	*PM0771*	membrane protein	1.49
1244119	*kpsS*	capsule polysaccharide transporter	1.47
1243460	*thrA*	bifunctional aspartokinase I/homoserine dehydrogenase I	1.46
1244484	*priA*	primosome assembly protein PriA	1.46
1244153	*PM0806*	ornithine decarboxylase	1.41
1243594	*cca*	CCA-adding protein	1.41
1244862	*pm1515*	hypothetical protein	1.40

1244098	*PM0751*	DNA glycosylase	1.39
1243668	*hscB*	CoA-transferase	1.36
1243588	*znuB*	zinc transporter	1.36
1245341	*lpxD*	UDP-3-O-(3-hydroxymyristoyl) glucosamine N-acyltransferase	1.35
1244128	*PM0781*	ATP-binding protein	1.33
1245059	*nanK*	N-acetylmannosamine kinase	1.33
1243730	*rnfG*	electron transporter RnfG	1.31
1243800	*PM0453*	iron permease	1.31
1244729	*speA*	arginine decarboxylase	1.30
1245131	*PM1784*	major facilitator transporter	1.27
1244067	*yfaE*	(2Fe-2S)-binding protein	1.27
1243477	*fecC*	iron ABC transporter	1.26
1245274	*PM1927*	D-alanyl-D-alanine carboxypeptidase	1.25
1244085	*PM0738*	hypothetical protein	1.25
1244343	*macB*	macrolide ABC transporter ATP-binding protein	1.24
1243666	*iscU*	scaffold protein, partial	1.23
1244265	*ubiC*	chorismate--pyruvate lyase	1.23
1244913	*PM1566*	hypothetical protein	1.22
1244651	*coaD*	phosphopantetheine adenylyltransferase	1.22
1244758	*rpsS*	30S ribosomal protein S19	1.22
1244090	*modB*	molybdate ABC transporter permease	1.22
1244565	*mutT*	nucleoside triphosphate pyrophosphohydrolase	1.21
1244783	*glpE*	thiosulfate sulfurtransferase	1.21
1244728	*PM1381*	agmatinase	1.19
1243541	*PM0194*	5'-methylthioadenosine/S-adenosylhomocysteine nucleosidase	1.19
1244510	*rnpA*	ribonuclease P	1.18
1244345	*ompV*	hypothetical protein	1.18
1244964	*PM1617*	hypothetical protein	1.18
1244116	*hrpA*	RNA helicase	1.17
1245095	*hslU*	ATP-dependent protease	1.17
1243489	*murG*	UDP-diphospho-muramoylpentapeptide beta-N-acetylglucosaminyltransferase	1.17
1243574	*secD*	preprotein translocase subunit SecD	1.16
1244338	*rne*	ribonuclease E	1.16
1243478	*fecB*	iron siderophore-binding protein	1.14
1244003	*tehB*	SAM-dependent methyltransferase	1.13
1244540	*ushA*	5'-nucleotidase	1.13
1243408	*rnc*	ribonuclease III	1.12
1243728	*PM0381*	endonuclease IV	1.12
1244897	*PM1550*	6-phosphogluconolactonase	1.12
1244840	*atpG*	F0F1 ATP synthase subunit gamma	1.11
1244841	*atpD*	F0F1 ATP synthase subunit beta	1.11

1243402	*menH*	2-succinyl-6-hydroxy-2,4-cyclohexadiene-1-carboxylate synthase	1.10
1244015	*fnr*	fumarate/nitrate reduction transcriptional regulator	1.10
1244141	*metC*	cystathionine beta-lyase	1.09
1245083	*rpoC*	DNA-directed RNA polymerase subunit beta'	1.09
1245047	*PM1700*	HAD family hydrolase	1.09
1244121	*hyaE*	protein HyaE	1.08
1243653	*PM0306*	CRISPR-associated protein Csy2	1.08
1245084	*rpoB*	DNA-directed RNA polymerase subunit beta	1.07
1244389	*eptA*	sulfatase	1.07
1243519	*lptA*	sugar ABC transporter substrate-binding protein	1.06
1243394	*nhaB*	sodium:proton antiporter	1.05
1244535	*tonB*	cell envelope protein TonB	1.05
1244511	*PM1164*	membrane protein	1.05
1243917	*tesB*	acyl-CoA thioesterase	1.04
1245246	*psd*	phosphatidylserine decarboxylase	1.04
1244901	*PM1554*	6-phosphogluconate dehydrogenase	1.04
1244230	*PM0883*	hypothetical protein	1.02
1245011	*ispH*	4-hydroxy-3-methylbut-2-enyl diphosphate reductase	1.02
1245133	*rseC*	hypothetical protein	1.02
1244512	*yidC*	membrane protein insertase	1.02
1244491	*rfaF*	hypothetical protein	1.01
1245096	*PM1749*	ATP-dependent protease	1.01

[a]: NCBI accession number of the identified gene.
