# Supplementary figures and images for: Identification of the crp gene in avian Pasteurella multocida and evaluation of the effects of crp deletion on its phenotype, virulence and immunogenicity
Source: BMC Microbiol. 2016 Jun 24;16:125. doi: 10.1186/s12866-016-0739-y (PMC4921010; doi:10.1186/s12866-016-0739-y)

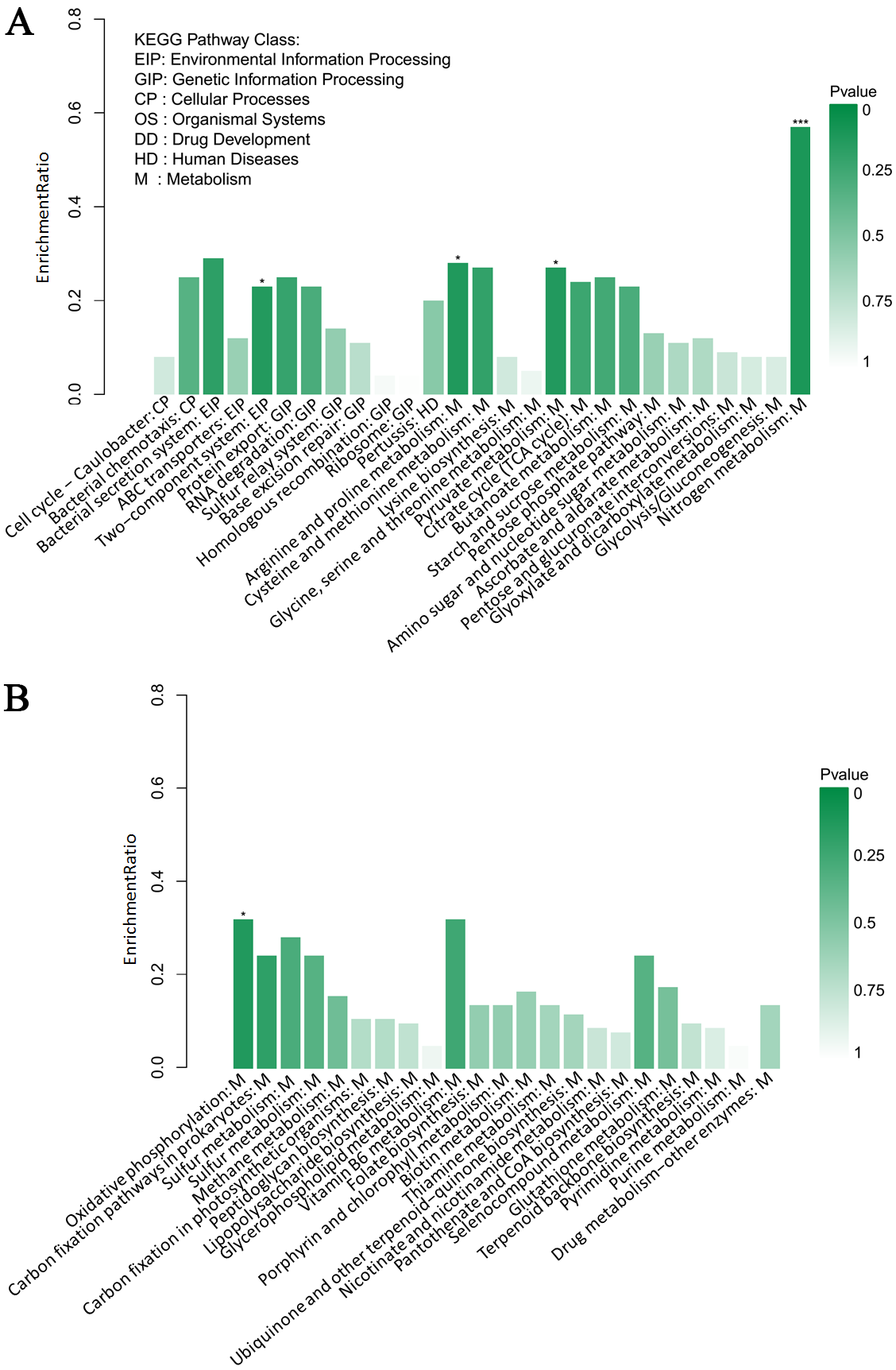

Supplement: Additional file 2: — KEGG enrichment analysis of the differentially expressed genes in the Δcrp mutant. KOBAS software was used to analyze the crp-regulated genes in KEGG pathways. Each column in A and B indicates one pathway, and the abscissa represents the name and classification of the pathway. The column color refers to the significance, and the depth of the color directly correlates with the degree of significance. *, p < 0.05; **, p < 0.01; ***, p < 0.001. (TIF 367 kb) [file 12866_2016_739_MOESM2_ESM.tif]
